# Small Molecule Activation by Two‐Coordinate Acyclic Silylenes

**DOI:** 10.1002/ejic.202000479

**Published:** 2020-07-28

**Authors:** Shiori Fujimori, Shigeyoshi Inoue

**Affiliations:** ^1^ Department of Chemistry WACKER‐Institute of Silicon Chemistry and Catalysis Research Center Technische Universität München Lichtenbergstraße 4 85748 Garching bei München Germany

**Keywords:** Silylenes, Acyclic compounds, Reaction mechanisms, Small molecule activation, Silicon

## Abstract

In recent decades, the chemistry of stable silylenes (R_2_Si:) has evolved significantly. The first major development in this chemistry was the isolation of a silicocene which is stabilized by the Cp* (Cp* = η^5^‐C_5_Me_5_) ligand in 1986 and subsequently the isolation of a first *N*‐heterocyclic silylene (NHSi:) in 1994. Since the groundbreaking discoveries, a large number of isolable cyclic silylenes and higher coordinated silylenes, i.e. Si(II) compounds with coordination number greater than two, have been prepared and the properties investigated. However, the first isolable two‐coordinate acyclic silylene was finally reported in 2012. The achievements in the synthesis of acyclic silylenes have allowed for the utilization of silylenes in small molecule activation including inert H_2_ activation, a process previously exclusive to transition metals. This minireview highlights the developments in silylene chemistry, specifically two‐coordinate acyclic silylenes, including experimental and computational studies which investigate the extremely high reactivity of the acyclic silylenes.

## Graphical abstract

Silylenes (:SiR_2_), the heavier analogues of carbenes (:CR_2_), have recently shown fascinating modes of reactivity, which are conventionally the domain of transition‐metal complexes. This minireview focuses on the developments in silylene chemistry, specifically two‐coordinate acyclic silylenes, including experimental and computational studies attempting to investigate the remarkable high reactivity of the acyclic silylenes.

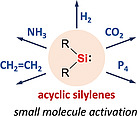

## 1. Introduction

The cleavage of rigid σ‐bonds, such as H–H bond, is a key step in a lot of important catalytic processes, conventionally the domain of transition metals.^1^ The ability of transition metals to bind reversibly with various functional groups enables transition‐metal complexes to perform as effective catalysts. In recent decades, the field of main‐group compounds has grown significantly and a variety of low‐valent main‐group compounds which show interesting reactivity have been reported.^2^ Silicon, the second most abundant element in the Earth's crust, is especially of interest due to its high natural abundance and low‐toxicity. Bond activation reactions using low‐valent silicon species in place of transition‐metal complexes are of importance, as the former are more environmentally friendly and cost‐effective than the latter. Over the past four decades, many low‐valent silicon compounds have been prepared using sterically demanding ligands (kinetic stabilization) and/or electronically stabilizing ligands based on heteroatom substituents (thermodynamic stabilization).^3^ Silylenes (:SiR_2_), the silicon analogues of carbenes (:CR_2_), have gained much attention due to their propensity to selectively activate small molecules.[2e] While the ground electronic state of carbenes (singlet or triplet) depends on the nature of the pendent substituents, silylenes generally exhibit a singlet ground state.^4^ The frontier molecular orbitals of singlet‐state silylenes consist of a high energy lone pair (HOMO) and an available vacant *p*‐orbital (LUMO). This dual donor/acceptor character (ambiphilicity) mimics the frontier *d*‐orbitals found in transition metals.[2a] As such, the activation of inert molecules (such as H_2_) using silylenes has been shown to be possible, a process previously exclusive to transition metals.

**Figure 1:**
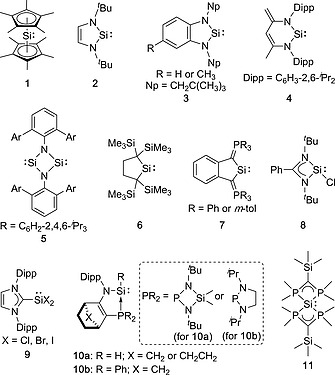
Selected examples of isolable silylenes stabilized by thermodynamic and/or kinetic stabilization effects.

In general, silylenes are of high reactivity, have short lifetimes and tend to undergo facile dimerization, oligomerization or polymerization. For example, silylenes bearing sterically bulky substituents such as Mes (Mes = 2,4,6‐Me_3_C_6_H_2_), dimerize to form the corresponding disilenes (R_2_Si=SiR_2_).^5^ Therefore, kinetic and/or thermodynamic stabilization is required to isolate such silylenes as stable compounds. One of important developments in main‐group chemistry was the isolation of a disilene Mes_2_Si=SiMes_2_ which was formed through the dimerization of the transient divalent silylene :SiMes_2_ at 77 K.^6^ Since the groundbreaking discovery, a variety of isolable cyclic silylenes and functionalized silylenes with a higher coordinated silicon(II) atom have been reported to date (Figure 1). One of the significant developments in the chemistry of stable silylenes is the isolation of the dodecamethylsilicocene :SiCp*_2_ (**1**) (Cp* = η^5^‐C_5_Me_5_) as a Si(II) compound with higher coordination number by Jutzi and co‐workers in 1986.^7^ Silicocene **1** is stabilized using the thermodynamic stabilization effect of the Cp* ligands. In 1994, Denk and co‐workers reported the first stable two‐coordinate *N*‐heterocyclic silylene (NHSi) **2**,^8^ which is the silicon analogue of the stable *N*‐heterocyclic carbene (NHC) isolated by Arduengo and co‐workers in 1991.^9^ Subsequent to this, the groups of Lappert and Gehrhus succeeded in isolating the benzo‐fused silylenes **3**.^10^ In 2006, the six‐membered NHSi **4** was described by Driess and co‐workers.^11^ Roesky and co‐workers reported the first base‐free bis‐silylene **5** in 2011.^12^ A large number of examples of NHSi's, which are stabilized by the effect of cyclic systems along with the interaction from the lone pairs on the directly bonded nitrogen atoms to the vacant 3p orbital on the silicon atom, were reported.^13^ In 1999, Kira and co‐workers succeeded in the synthesis of the first isolable cyclic dialkylsilylene **6** using the kinetic stabilization effect by the sterically bulky dialkyl based helmet‐type ligand.^14^ Furthermore, Driess and co‐workers reported the synthesis of carbocyclic silylenes **7** bearing two phosphonium ylides which exhibit comparable aromatic character.^15^

Apart from these cyclic silylenes, a number of Lewis base stabilized silylenes have been prepared.^16^ In this decade, many studies on three coordinate Si(II) compounds have been demonstrated and their interesting electronic features and fascinating reactivity have been revealed. A significant discovery in this field was the synthesis of three coordinate silylenes bearing halogens [:SiX_2_(L)] (X = halogen) which are widely used as precursors to synthesize novel silicon compounds. In 2006, the first example of isolable monomeric chlorosilylene (:SiCl[PhC(N^*t*^Bu)_2_]) **8** stabilized by an amidinate ligand was reported by Roesky and co‐workers.^17^ NHC‐stabilized dihalosilylenes [:SiX_2_(NHC) (X = halogen)] **9** are also indispensable building blocks in synthetic chemistry.[16c], 18 Another remarkable recent achievement in such three coordinate systems are the preparation of hydrosilylenes [:Si(H)R] which are attractive compounds for applications in catalytic transformations such as the hydrosilylation of alkenes, alkynes and carbonyl compounds. There are only a few examples of isolated hydrosilylenes without using Lewis acid stabilization.^19^ Kato and Baceiredo reported the isolation of a three coordinate Si(II) hydride **10a** which is stabilized by an intramolecular phosphine coordination.[19a] Similarly, the phenyl‐substituted silylene **10b** stabilized by a similar phosphine based ligand was isolated.^20^ In addition, four coordinate silicon(II) compounds, e.g. tetraphosphorus coordinated Si(II) compound (**11**), have been reported.^21^ While many isolable cyclic silylenes^13^, ^22^ and Lewis base stabilized silylenes^16^ have been described to date, only a few examples of simple dicoordinate acyclic silylenes are known, because the isolation of such silylenes as stable compounds is synthetically challenging due to their highly reactive nature.

**Figure 2:**
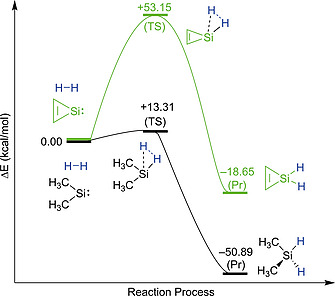
The activation energy and the exothermic energy for the H_2_ insertion reaction with the acyclic silylene (black line) and the cyclic silylene (light green line), calculated at the B3LYP/6‐311+G** level of theory.^23^ Transition state (TS), addition product (Pr).

In a previous theoretical study, Wang and Ma investigated the small molecule activation, specifically H_2_, to the variety of cyclic and acyclic silylenes.^23^ Some of the key factors which influence the reactivity of silylenes towards H_2_ activation are the HOMO–LUMO and singlet–triplet gaps. In the case of NHSi's which exhibit large HOMO–LUMO and singlet–triplet energy gaps, high activation energies are required to reach the corresponding product. Another important factor in the reaction behavior of silylenes is the geometry around the silicon center, especially the angle at the silicon atom. When the ring strain is large, a higher barrier is required in breaking a H_2_ molecule. For instance, the activation energy for the three‐membered silylene ring, silacyclopropenylidene (53.15 kcal/mol), is much higher compared with that of the acyclic dimethylsilylene (:SiMe_2_) (13.31 kcal/mol) due to the ring strain along with the 2π‐electrons‐delocalization on the C–C–Si ring (Figure 2). Similarly, in the case of nitrogen‐substituted systems, the higher activation barrier (63.46 kcal/mol) for the *N*‐heterocyclic silylene is required to reach the product than that (45.59 kcal/mol) of the diaminosilylene [:Si(NH_2_)_2_].^23^ It is also found that the splitting of H_2_ with acyclic silylenes is more exothermic (–50.89 kcal/mol for dimethylsilylene, –23.34 kcal/mol for diaminosilylene) than that of cyclic silylenes (–18.65 kcal/mol for silacyclopropenylidene, –6.65 kcal/mol for *N*‐heterocyclic silylene). In a recent computational study, Kuriakose and Vanka investigated the single site small molecule activation by acyclic silylenes and the undesired side reaction.^24^ In these systems during H_2_ splitting the undesired side reaction, which leads to decomposition of the silylenes forming the products :Si(H)R' and HR (decomposition reaction), would be competitive to the desired reaction in which both hydrogen atoms bind to the same atom to form the tetravalent RSi(R')H_2_ product (single site reaction) (R/R' = thiolato/thiolato, boryl/amido, or silyl/amido). The study indicated that the angle at the silicon center also affects the preference of silylenes for the single site or the decomposition pathway. When the angle becomes even smaller, the dissociation pathway is favored over the single site pathway significantly.

In the last three decades, the chemistry of stable silylenes has grown significantly and has been subject to many recent reviews regarding isolable cyclic silylenes,^13^, ^22^ functionalized silylenes with higher coordinate silicon(II) centers,^16^ and their application towards small molecule activation and catalytic reactions.[2c], 25 Despite recent progress, the activation of inert molecules such as H_2_ using silylenes remains scarce. The computational studies implied that acyclic silylenes with a highly obtuse angle at the silicon center would have a low‐lying triplet excited state and allow for the activation of inert molecules. Therefore, the study of such silylenes may open new doors to the reactivity of main‐group compounds as transition metal mimics. The focus of this minireview is the properties and key reactivity highlights from simple dicoordinate acyclic silylenes.

## 2. Synthesis

**Scheme 1:**
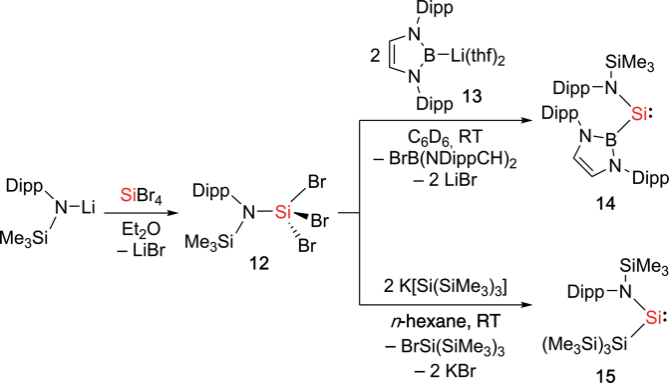
Synthesis of amido(boryl)silylene **14** and amido(silyl)silylene **15**.

**Scheme 2:**
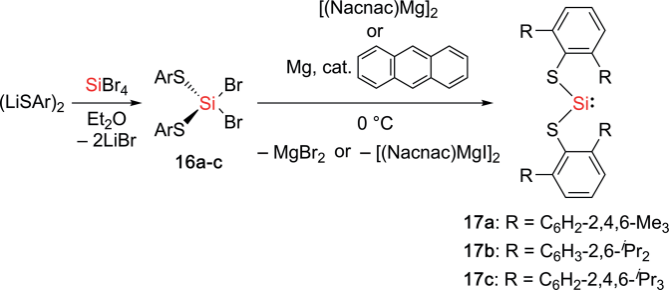
Synthesis of bis(arylthiolato)silylenes **17a–c**.

**Scheme 3:**
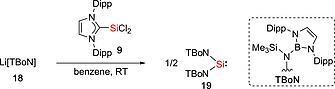
Synthesis of bis(amido)silylene **19**.

**Scheme 4:**
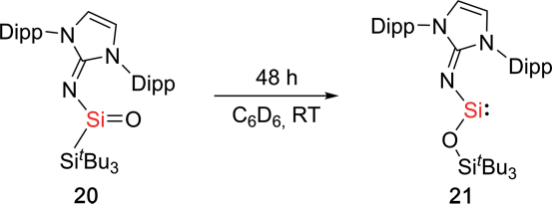
Synthesis of imino(siloxy)silylene** 21**.

**Scheme 5:**
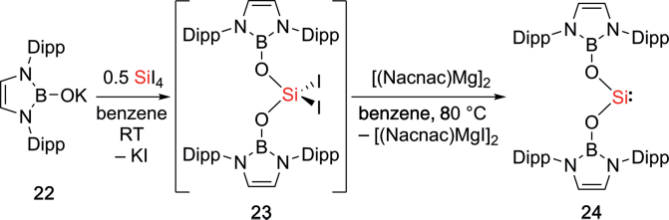
Synthesis of bis(boryloxy) silylene **24**.

**Scheme 6:**
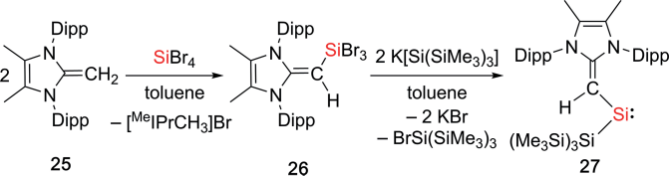
Synthesis of vinyl(silyl)silylene **27**.

Although a variety of isolable cyclic and Lewis base stabilized silylenes have been studied, only a handful of isolable dicoordinate acyclic silylenes have been reported. While diamino substituted acyclic silylenes have been observed by NMR and UV/Vis spectroscopy, these compounds are unstable at ambient temperature.^26^ The first examples of isolable dicoordinate acyclic silylenes **14** and **17a** were reported in 2012.^27^, ^28^ (Scheme 1 and Scheme 2). The sterically demanding boryl‐substituted silylene :Si{B(NDippCH)_2_}{N(SiMe_3_)‐Dipp} (**14**) (Dipp = 2,6‐^*i*^Pr_2_C_6_H_3_), was prepared by the reaction of a tribromo(amino)silane **12** with two equivalents of lithiumboryl reagent **13**.^27^ Alternatively, **12** reacts readily with two equivalents of hypersilylpotassium [K(THF)_2_][Si(SiMe_3_)_3_] to give the corresponding silyl‐substituted silylene :Si{Si(SiMe_3_)_3_}{N(SiMe_3_)‐Dipp} (**15**) (Scheme 1).^29^ In the solid state, both silylenes **14** and **15** exhibit remarkable thermal robustness (*T*_d_ = ca. 140 °C for **14** and **15**). The synthesis of an extensive series of arylthio substituted silylenes **17a**–**c** was reported (Scheme 2).^28^, ^30^ Bis(arylthiolato)silylene :Si(SAr^Me6^)_2_ (**17a**) [Ar^Me6^ = 2,6‐(2,4,6‐Me_3_C_6_H_2_)_2_C_6_H_3_] was synthesized by the reduction of Br_2_Si(SAr^Me6^)_2_ **17a** with Jones' Mg(I)–Mg(I) complex [(Nacnac)Mg]_2_ {Nacnac = HC‐[(Me)CN(Mes)]_2_}.^28^ Silylene **17a** was found to be stable up to 146 °C. However, attempts to synthesize the silylenes :Si(SAr^*i*Pr4^)_2_ (**17b**) [Ar^*i*Pr4^ = 2,6‐(2,6‐^*i*^Pr_2_C_6_H_3_)_2_C_6_H_3_] and :Si(SAr^*i*Pr6^)_2_ (**17c**) [Ar^*i*Pr6^ = 2,6‐(2,4,6‐^*i*^Pr_3_C_6_H_2_)_2_C_6_H_3_] were unsuccessful due to the bulkier substituents.^30^ Alternatively, the reduction of the dibromobisthiolato Si(IV), Br_2_Si(SAr^*i*Pr4^)_2_ **16b** and Br_2_Si(SAr^*i*Pr6^)_2_ **16c**, with Rieke's magnesium^31^ and a catalytic amount of anthracene afforded the silylenes :Si(SAr^*i*Pr4^)_2_ (**17b**) and :Si(SAr^*i*Pr6^)_2_ (**17c**). The attempt to synthesize the silylene :Si(SAr^Me6^)_2_ (**17a**) by the reduction using only Rieke's magnesium was unsuccessful. Only amide substituted silylene :Si(TBoN)_2_ (**19**) stabilized by two extremely bulky boryl‐amido ligands, [N(SiMe_3_){B(DAB)}]^–^ [TBoN; DAB = (DippNCH)_2_], was synthesized by the groups of Jones and Aldridge (Scheme 3).^32^ The reaction of Li[TBoN] **18** with the dichlorosilylene :SiCl_2_(IPr) (**9**) [IPr = :C(HCNDipp)_2_] gave a mixture of the silylene :Si(TBoN)_2_ **19** and IPr in a ratio of 1:1. Silylene **19** is thermally stable both in the solid state (m.p. 152–160 °C) and in hydrocarbon solutions at room temperature. Recently, our group succeeded in isolating the first example of acyclic neutral silanone which contain a planar three‐coordinate Si atom.^33^ Interestingly, while silanone **20** is stable in the solid state at ambient temperature, a solution (C_6_D_6_ or [D_8_]THF) of **20** is fully converted into the imino(siloxy)silylene :Si(OSi^*t*^Bu_3_)(IPrN) (**21**) at room temperature within 48h (Scheme 4). The Aldridge group reported the bis(boryloxy) silylene :Si[(HCDippN)_2_BO]_2_ (**24**) (Scheme 5). The reaction of **22** with half an equivalent of SiI_4_ furnished the intermediate [(HCDippN)_2_BO]_2_SiI_2_ **23**. Subsequent reduction of **23** with Jones' reagent at 80 °C resulted in the formation of the bis(boryloxy) silylene **24** which is thermally stable at 80 °C over several days.^34^ The Rivard group utilized a bulky vinylic ligand [(^Me^IPr)CH]^–^, (^Me^IPr = (MeCNDipp)_2_C) to generate the vinyl(silyl)silylene **27** stabilized by a carbon‐based donor. The treatment of a toluene solution of (^Me^IPrCH)SiBr_3_ (**26**) with two equivalents of [K(THF)_2_][Si(SiMe_3_)_3_] afforded the silylene **27** (Scheme 6).^35^ Compound **27** is remarkably stable both in the solid state (m.p. 110–112 °C) and in benzene solution at ambient temperature for a couple of months.

**Scheme 7:**
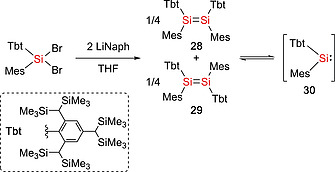
Dynamic equilibrium between disilenes (**28** and **29**) and silylene **30**.

**Scheme 8:**
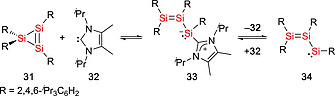
Equilibrium of cyclotrisilene **31** and *N*‐heterocyclic carbene **32** with NHC‐coordinated disilenyl silylene **33**.

**Scheme 9:**
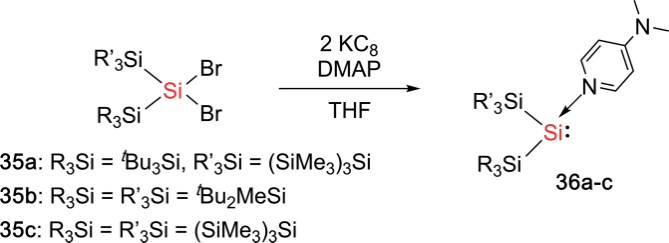
Synthesis of DMAP‐stabilized silylenes **36a–c**.

**Scheme 10:**
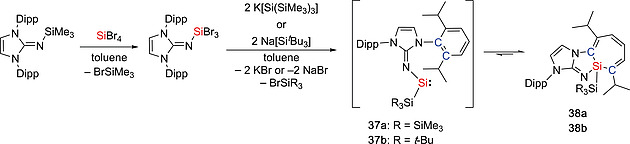
Dynamic equilibria between silepins **38a–b** and imino(silyl)silylenes **37a–b**.

**Scheme 11:**
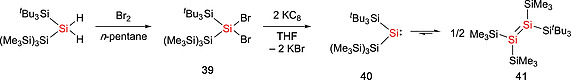
Dynamic equilibrium between disilene **41** and bis(silyl)silylene **40**.

Some disilenes (R_2_Si=SiR_2_) are in equilibrium with their monomeric silylene (:SiR_2_) form in solution and are therefore synthetically equivalent to silylenes.^36^, ^37^ The dissociation energy of the Si=Si double bond in disilenes depends on the singlet–triplet energy gap of the corresponding monomer, therefore the nature of substituents at the silicon atom play important roles. Whilst electropositive and π‐accepting substituents stabilize the triplet state and the Si=Si double bond in the disilenes, electronegative and π‐donating substituents stabilize the singlet and induce to the dissociation of disilenes into the corresponding silylenes.^36^ Additionally, in some disilenes, the Si=Si bond is weakened by the steric repulsion between the sterically bulky substituents, leading to the dynamic disilene‐silylene equilibria.^37^ The first example of the disilene‐silylene equilibrium was reported in 1993.^38^ Disilenes Tbt(Mes)Si=Si(Mes)Tbt (**28**, **29**) (Tbt = 2,4,6‐[CH(SiMe_3_)_2_]_3_C_6_H_2_) bearing sterically bulky substituents was found to undergo thermal dissociation which resulted in the formation of the corresponding silylene :Si(Mes)Tbt (**30**) (Scheme 7). It was found that the silylene :Si(Mes)Tbt (**30**) can be trapped with aryl isocyanides ArCN [Ar = Tbt, 2,4,6‐(CHMe_2_)_3_C_6_H_2_, or 2,4,6‐^*t*^Bu_3_C_6_H_2_] to give the corresponding silylene‐isocyanide complexes [Tbt(Mes)Si‐CNAr], which are the first isolable silylene‐Lewis base complexes and can act as a masked silylene.[38c] In addition, some other silicon compounds behave as masked silylenes.^39^ The groups of Scheschkewitz and Rzepa reported the isolation of a disilenyl silylene stabilized by an NHC (**33**) which coexists in equilibrium with the isomeric cyclotrisilene **31** and the free NHC **32** in solution (Scheme 8).[39a], [39b] With regard to this field, our group has demonstrated that DMAP‐stabilized silylenes, :Si(SiR_3_)(SiR'_3_)(DMAP) [**36a**: SiR_3_ = Si^*t*^Bu_3_, SiR'_3_ = Si(SiMe_3_)_3_, **36b**: SiR_3_ = SiR'_3_ = SiMe^*t*^Bu_2_, **36c**: SiR_3_ = SiR'_3_ = Si(SiMe_3_)_3_] (DMAP = 4‐*N,N*‐dimethylaminopyridine), act as masked silylenes and react with small molecules such as H_2_ and ethylene at 65 °C (Scheme 9).[39c] In addition, the dynamic equilibria between silepins **38a**–**b** and imino(silyl)silylenes :Si(IPrN){Si(SiMe_3_)_3_} (**37a**) and :Si(IPrN)(^*t*^Bu_3_) (**37b**) are revealed by experimental and computational studies (Scheme 10).^33^, ^40^ The intramolecular insertion reaction of the in situ generated silylenes **37a** and **37b** into the C=C bond of the aromatic ligand framework resulted in the formation of silepins **38a** and **38b**. Reactivity studies revealed that silepin **38a**, serving as silylene **37a** in situ, is capable of the activation of small molecules (vide infra). Our group also presented an isolable bis(silyl)silylene **40** which is in equilibria with the corresponding tetrasilyldisilene **41**. The reaction of dibromosilane {(TMS)_3_Si}(^*t*^Bu_3_Si)SiBr_2_ (**39**) with KC_8_ afforded an equilibrium mixture of **40** and **41** (Scheme 11).^41^ These compounds **38** and **41** can behave as synthetic alternatives to acyclic silylenes.

## 3. Properties

Several examples of stable two‐coordinate acyclic silylenes have been structurally characterized using single‐crystal X‐ray diffraction analysis. The information can help to explain the compound's reactivity and stability. Selected structural parameters of these acyclic silylenes are shown in Table 1.

**Table 1:** Selected structural parameters, spectroscopic data, and HOMO–LUMO and singlet–triplet energy gaps for the acyclic silylenes

	∠E–Si–E'/°	*d*(Si–E)/Å	^29^Si chemical shifts (*δ*)	*λ*_max_[nm]	HOMO–LUMO gap [eV]	Singlet–triplet gap (kJ/mol)
						
:Si{B(NDippCH)_2_}{N(SiMe_3_)‐Dipp} (**14**)	B–Si–N	Si–B: 2.066(1)	+439.7	–	2.04^a^	103.9^a^
	109.7(1)	Si–N: 1.731(1)				
:Si(SAr^Me6^)_2_ (**17a**)	S–Si–S	Si–S: 2.1607(5)	+285.5	382	4.26^b^	–
	90.519(19)	Si–S: 2.1560(5)				
:Si(SAr^*i*Pr4^)_2_ (**17b**)	S–Si–S	Si–S: 2.137(1)	+270.4	405	–	–
	85.08(5)	Si–S: 2.137(1)				
:Si(SAr^*i*Pr6^)_2_ (**17c**)	S–Si–S	Si–S: 2.089(9)^c^	+270.9	411	–	–
	84.8(1)					
:Si{Si(SiMe_3_)_3_}{N(SiMe_3_)‐Dipp} (**15**)	N–Si–Si	Si–Si: 2.386(1)	+438.2	–	1.99^a^	103.7^a^
	116.91(5)	Si–N: 1.720(1)	+467.5			
:Si(TBoN)_2_ (**19**)	N–Si–N	Si–N: 1.7495(10)	+204.6	385	2.55^d^	158.3^d^
	110.94(5)	Si–N: 1.7432(10)				
:Si(OSi^*t*^Bu_3_)(IPrN) (**21**)	N–Si–O	Si–N: 1.661(2)	+58.9	328	4.33^e^	–
	103.56(8)	Si–O: 1.643(1)				
:Si[(HCDippN)_2_BO]_2_ (**24**)	O–Si–O	Si–O: 1.6074(14)	+35.5	348	5.45^f^	–
	100.02(8)	Si–O: 1.6052(14)				
:Si(^Me^IPrCH){Si(SiMe_3_)_3_} (**27**)	C–Si–Si	Si–C: 1.798(2)	+432.9	583	4.79^g^	–
	101.59(7)	Si–Si: 2.404(1)				
:Si(IPrN){Si(SiMe_3_)_3_} (**37a**)	–	–	+300.0	612	2.96^h^	103.9^h^
:Si(Si^*t*^Bu_3_){Si(SiMe_3_)_3_} (**40**)	–	–	–	–	4.18^i^	10.5^i^
:Si[Ga(Br)L]_2_ (**46**)	–	–	–	–	2.7^j^	5.9^j^

a Calculated at the PBE/TZ2P level of theory.

b Calculated at the PBE1PBE/def2‐TZVP level of theory.

c Averaged value.

d Calculations at the BP86/TZP level of theory were performed on the molecule with its Dipp substituents replaced by 2,6‐dimethylphenyl groups.

e Calculated at the B3LYP/6‐311+G(d) level of theory.

f Calculated at the PBE1PBE/TZVP level of theory.

g Calculated at the M06‐2X/def2‐TZVP level of theory. See the reference.^42^

h Calculations at the B3LYP/6‐311+G(d,p) level of theory were performed on the molecule with its Dipp substituents replaced by phenyl groups.

i Calculated at the M06‐2X/6‐31+G(d,p) level of theory.

j Calculated at the B3LYP‐D3(BJ)/6‐311G(d,p), def2‐TZVP (Ga and Br) level of theory.

The angles for the silicon centers in silylenes **14**, **15**, and **19** [109.7(1)° (**14**), 116.91(5)° (**15**), 110.94(5)° (**19**)] are wider than those in NHSi's [86.02(6)°–99.31(5)°].^8^, ^10^, ^11^, ^12^, ^13^ Silylenes bearing electronegative siloxy (**21**) and boryloxy (**24**) substituents and the vinyl(silyl)silylene (**27**) exhibit narrower bond angles [103.56(8)° (**21**); 100.02(8)° (**24**); 101.59(7)° (**27**)] than those found in other reported acyclic silylenes. These results suggested the presence of a high degree of *s*‐character at the lone pair of the silicon center in silylenes. While the angles for the silicon center in the imino(siloxy)silylene **21** and the bis(boryloxy)silylene **24** are similar to each other, the Si–O distances in **24** [1.6074(14) Å and 1.6052(14) Å] are relatively shorter than that in **21** [1.643(1) Å]. This is likely because of the more strongly electron‐donating NHI (*N*‐heterocyclic imine) ligand leading to the dominant *p*‐donor contribution in **21** while **24** has two O‐donor ligands. Owing to oxygen's small atomic radius, the obtuse O–Si–O angle in the bis(boryloxy)silylene **24** [100.02(8)°] is wider than the S–Si–S angles in the bis(arylthiolato)silylenes **17a–c** [90.52(2)° (**17a**), 85.08(5)° (**17b**), 84.8(1)° (**17c**)] due to the increased steric repulsion between the bulky boryloxy ligands and charge repulsion between the lone pairs. Additionally, the geometry of silylenes has an effect on the HOMO–LUMO energy, as silylenes which have wider E–Si–E' angles tend to exhibit smaller HOMO–LUMO energy gaps (Table 1). Previous computational studies imply silylenes which exhibit small HOMO–LUMO gaps and coordinative flexibility should be ideal for selective activation of relatively unreactive small molecules.

Further information regarding the chemical bonding in silylenes is gained by the ^29^Si NMR spectrum (Table 1). The ^29^Si NMR chemical shifts for the two‐coordinate silicon center of the acyclic bis(amido)silylene **19** (+204.6 ppm) is downfield shifted relative to those in NHSi's (*δ* = 78–119 ppm).^43^ In the case of NHSi's, the lone pairs on the nitrogen atoms are parallel to the empty 3p orbital on the silicon atom leading to an efficient π‐overlap and increased shielding of the silylene resonance. Similarly, bis(arylthiolato)silylenes **17a–c** and imino(silyl)silylene **37a** show a downfield shift in the ^29^Si NMR spectrum [+285.5 (**17a**), +270.4 (**17b**), +270.9 (**17c**), +300.0 (**37a**) ppm]. These results suggested much less π‐donation from the sulfur or nitrogen atoms to the vacant *p* orbital on the silicon atom relative to that in NHSi's. Furthermore, amido(boryl)silylene **14**, amido(silyl)silylene **15**, and vinyl(silyl)silylene **27**, which have the wider E–Si–E' angle [101.59(7)°–116.91(5)°] compared with those of the bis(arylthiolato)silylenes **17a**–**c** [84.8(1)°–90.52(2)°] and NHSi's [86.02(6)°–99.31(5)°], show an enormous downfield signal [+439.7 (**14**), +438.2, +467.5 (**15**), +432.9 (**27**)] in the ^29^Si NMR spectrum. The results indicate a significantly large electrophilicity of the divalent silicon center which is reminiscent of that observed in the dialkyl‐substituted cyclic silylene **6** (567.4 ppm).^14^ The ^29^Si NMR spectrum of **21** and **24** feature a significantly highfield resonance [+58.9 (**21**), +35.5 (**24**) ppm] compared to other dicoordinate acyclic silylenes, which suggests additional π‐donation by the siloxy or boryloxy ligands.

## 4. Small Molecule Activation with Acyclic Silylenes

As mentioned in the introduction, low‐valent main‐group compounds such as silylenes bearing a high energy HOMO and an energetically accessible LUMO can mimic the reactivity of transition metal complexes. The ability of transition metals to facilitate the activation of small molecules (H_2_, CO, alkenes etc.) has enabled the widespread development of homogeneous transition metal catalysis. Recently, key catalytic reaction steps (oxidative addition, insertion reactions, reductive elimination) have been reported for the low‐valent main‐group compounds.^2^ However, the cleavage of rigid σ‐bonds such as H–H by main‐group compounds remain scarce. Silylenes are of extremely high reactivity due to the high‐energy lone‐pair on silicon (HOMO) and the low‐lying vacant *p* orbital (LUMO) that enable to activate these small molecules. Acyclic silylenes are expected to exhibit high reactivity relative to their cyclic counterparts due to their wide E–Si–E' angles and small HOMO–LUMO gaps. In this section, the small molecule activation by two‐coordinate acyclic silylenes is outlined.

### 4.1 Activation of H_2_

The cleavage of dihydrogen is a key step in numerous homogeneous catalytic processes such as the hydrogenation of unsaturated organic compounds and hydroformylation reactions.^44^ Additionally, the adsorption/regeneration of H_2_ is important processes in potential hydrogen storage materials.^45^ This desirable reactivity towards H_2_ is generally mediated by transition metals, however it has recently been demonstrated that reduced main‐group centers exhibit this reactivity as well.

**Scheme 12:**
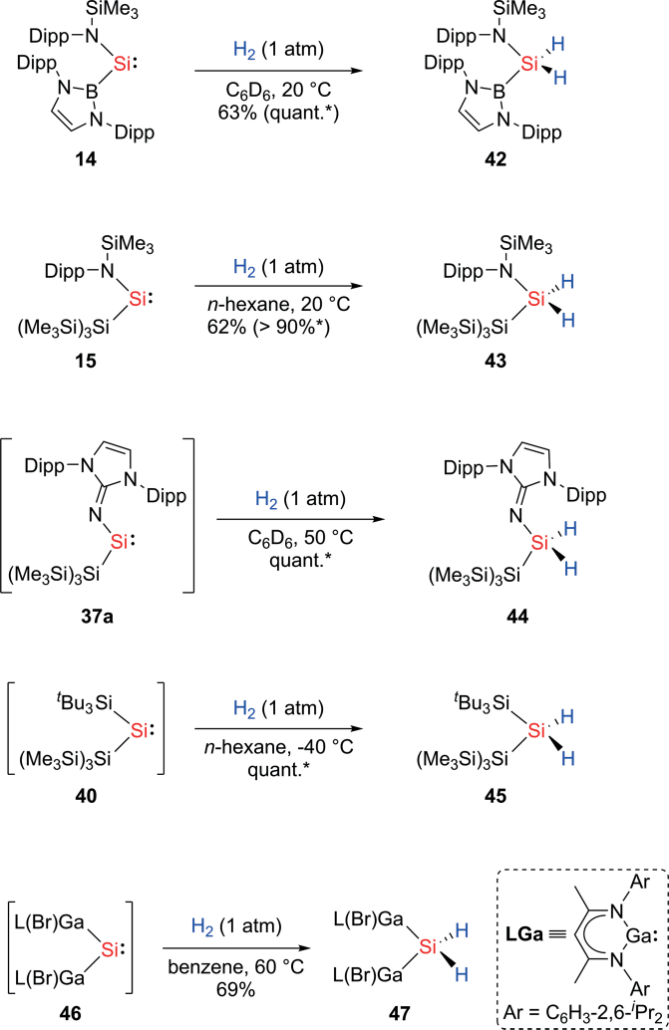
Activation of H_2_ by acyclic silylenes. *Estimated yield by NMR spectroscopy.

Previous theoretical studies on the reactivity of a variety of cyclic and acyclic silylenes towards the H_2_ activation revealed that the electronic structure features (HOMO–LUMO or singlet–triplet energy gaps) in these silylenes have effect on the accessibility of H_2_ activation.^23^ *N*‐Heterocyclic silylenes, bis(arylthiolato)silylene :Si(SAr^Me6^)_2_ (**17a**),^28^ bis(amido)silylene :Si(TBoN)_2_ (**19**),^32^ and imino(siloxy)silylene :Si(OSi^*t*^Bu_3_)(IPrN) (**21**)^33^ have shown no reaction toward H_2_ due to their large HOMO–LUMO gaps (and its heavily sterically protected silylene center). The first example of the H_2_ activation with a silylene was reported in 2012. Amido(boryl)silylene :Si{B(NDippCH)_2_}{N(SiMe_3_)‐Dipp} (**14**) was found to undergo H_2_ activation at mild conditions, affording the dihydrosilane H_2_Si{B(NDippCH)_2_}{N(SiMe_3_)‐Dipp} (**42**) (Scheme 12).^27^ Similarly, amido(silyl)silylene :Si{Si(SiMe_3_)_3_}{N(SiMe_3_)‐Dipp} (**15**) has shown reaction towards H_2_ at ambient temperatures to yield the corresponding dihydrosilane H_2_Si{Si(SiMe_3_)_3_}{N(SiMe_3_)‐Dipp} (**43**).^29^ More recently, our group performed the activation of H_2_ with the *N*‐heterocyclic imino‐ligated silepin **38a**, serving as silylene **37a** in situ, which resulted in the formation of the corresponding dihydrosilane **44**.^40^ The π‐donating substituents (amido or NHI ligands) in silylenes **14**, **15**, and **37a** lead to a decreased HOMO–LUMO gap [2.04 eV (**14**), 1.99 eV (**15**), 2.96 eV (**37a**)], which allows for the activation of inert molecules. Furthermore, the disilene **41**/silylene **40** equilibrium mixture was also found to activate H_2_ under very mild conditions (–40 °C).^41^ Interestingly, the HOMO–LUMO energy gap in the singlet bis(silyl)silylene **40** (4.18 eV) is comparably larger than those of acyclic silylenes, which are able to activate H_2_, and similar to those of acyclic silylenes **17a** (4.26 eV) and **21** (4.33 eV), which have shown no reaction toward H_2_. The singlet–triplet energy gap (10.5 kJ/mol) for bis(silyl)silylene **40** is small due to the effect of the electropositive bulky silyl substituents. Very recently, the groups of Schulz and Schreiner reported H_2_ splitting by a silylene intermediate :Si[Ga(Br)L]_2_ (L = HC[C(Me)N(2,6‐^*i*^Pr_2_C_6_H_3_)]_2_) (**46**).^46^ The treatment of [L(Br)Ga]_2_SiBr_2_ with an equimolar amount of LGa at 60 °C under H_2_ resulted in the formation of the dihydrosilane H_2_Si[Ga(Br)L]_2_ (**47**). Silylene **46** exhibits the lowest HOMO–LUMO gap energy (2.7 eV) and smallest singlet–triplet gap (5.9 kJ/mol).

### 4.2 Activation of NH_3_

Although many examples of H_2_ activation by transition metal complexes have been reported, N–H bond activation is more challenging as Werner‐type complexes are readily formed in the reaction with Lewis basic amines.^47^ The activation of the N–H bonds of ammonia has attracted attention for applications in catalytic transformations such as hydroamination. While very few of examples of N–H bond activation by transition metal complexes are known, it has been revealed that many low‐valent main‐group compounds undergo such activation processes.[47b]

**Scheme 13:**
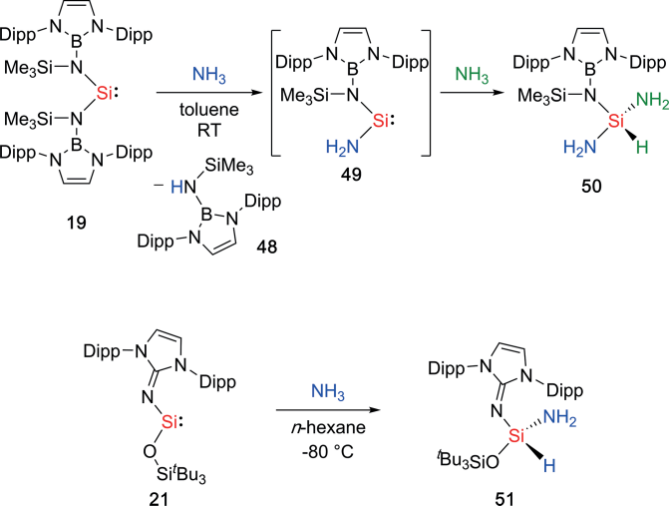
Activation of NH_3_ by acyclic silylenes.

Bis(amido)silylene **19** reacts with NH_3_ to yield triaminosilane **50** together with the secondary amine TBoNH (**48**) (Scheme 13).^32^ The plausible mechanism involves the formation of diaminosilylene **49** via a σ‐bond metathesis reaction between **19** and NH_3_, followed by the oxidation addition to NH_3_ to afford the triaminosilane **50**.^48^ This observation is in agreement with a previously computed σ‐bond metathesis H_2_ activation pathway mediated by silylene.^24^ Recently, our group reported the reactivity of the imino(siloxy)silylene **21**.^49^ The reaction of silylene **21** with 1 equivalent of NH_3_ affords the hydroamination product **51**. It is of note that compound **51** even reacts with excessive amounts of NH_3_ to yield an unidentified mixture. In the reaction, IPrNH and (H_2_N)(^*t*^Bu_3_SiO)Si(H)(NH_2_) were formed, similar σ‐bond metathesis reaction to that observed for the bis(amido)silylene **19**.

### 4.3 C–O Bond Activation

Carbon dioxide is a potent greenhouse gas in the atmosphere and a versatile feedstock for chemical or material production.^50^ To date, many studies on carbon capture and storage (CCS) of CO_2_ along with its chemical activation and utilization as a C1 source have been demonstrated. While transition metals have been utilized in most CO_2_ activation, the development of transition‐metal free and eco‐friendly systems have been underexplored. Currently, some low‐valent silicon compounds which undergo CO_2_ activation have been reported.^51^

**Scheme 14:**
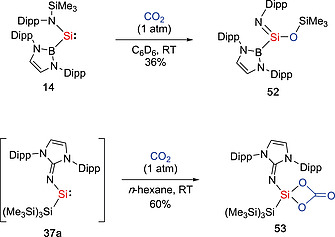
Activation of CO_2_ by acyclic silylenes.

**Scheme 15:**
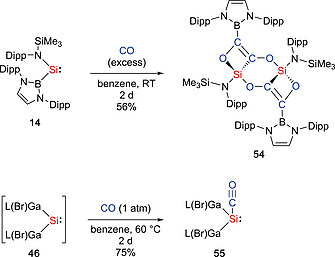
Activation of CO by acyclic silylenes.

Our group found that silepin **38a**, which behaves as dormant form of imino(silyl)silylene **37a**, rapidly reacts with CO_2_ under mild conditions (1 atm, r.t., within 1h) to afford the corresponding silicon carbonate **53** (Scheme 14).^40^ Comparable to mechanisms described in literature,[51e], 52 it is plausible that the transient silanone (O=Si(IPrN){Si(SiMe_3_)_3_}) was formed by the oxidative addition of CO_2_ and extrusion of CO, followed by the cycloaddition of another molecule of CO_2_. While such compounds tend to dimerize,^51^ our group demonstrated that the isolation of the first four‐coordinate, monomeric silicon carbonate **53** in high yields. The reaction of **14** with CO_2_ under mild conditions (1 atm, r.t.) resulted in the formation of the (trimethylsiloxy)iminosilane {(HCDippN)_2_B}Si(NDipp)(OSiMe_3_) (**52**).^53^ It is plausible that the in situ generation of the silanone [O=Si{B(NDippCH)_2_}{N(SiMe_3_)‐Dipp}],^54^ followed by silyl group migration than the bimolecular reaction with CO_2_ yields the carbonate.^55^ Silylene **14** also reacts with CO at ambient temperature to yield **54** which was characterized by standard spectroscopic techniques and X‐ray crystallographic analysis (Scheme 15).^53^ Compound **54** contains two Si(IV) centers, which bind to one carbon and two oxygen atoms derived from CO, along with the amide ligand. Although the activation of carbon monoxide and formation of the stable carbonyl complexes under mild condition is well known for transition‐metal complexes, such reaction is virtually unknown for main‐group compounds. Recently, the groups of Schulz and Schreiner reported the isolation of the silylene carbonyl complex [L(Br)Ga]_2_Si:–CO (**55**) (L = HC[C(Me)N(2,6‐^*i*^Pr_2_C_6_H_3_)]_2_). The reaction of GaL with SiBr_4_ under a CO atmosphere, generates the silylene [L(Br)Ga]_2_Si: (**46**) in situ, subsequently affording the silylene carbonyl complex **55**.^46^ Compound **55** is remarkably stable both in the solid state (*T*_d_ = 176–177 °C) and in solution, no decomposition was observed in toluene solution up to 80 °C. Furthermore, silylene carbonyl complex **55** acts as a masked silylene and reacts with H_2_ to give the dihydrosilane H_2_Si[Ga(Br)L]_2_ (**47**).

### 4.4 C=C and C≡C Bonds Activation

The activation of small organic molecules and the formation of C–C bonds is a fundamentally important process for transformation of simple molecules into essential chemical compounds in both academia and industry.^56^ For this purpose, transition metal catalysts have been utilized and a large number of useful catalysts have been developed. On the one hand, the catalytic bond activation and C–C bond formation are challenging for main group compounds as their oxidation states vary in a much narrower range. Recently, some main group element compounds have shown the activation of C–C bonds in neutral organic molecules such as alkenes alkynes as well as its dynamic equilibrium that is a key step in catalytic processes. Furthermore, it was demonstrated that catalytic activation of alkynes, followed by the formation of C–C bonds by utilizing low‐valent main‐group compounds is also possible.^57^

**Scheme 16:**
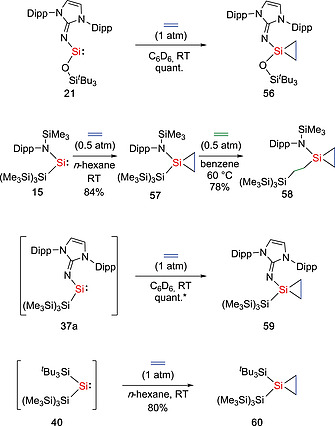
Activation of ethylene by acyclic silylenes. *Estimated yield by NMR spectroscopy.

**Scheme 17:**
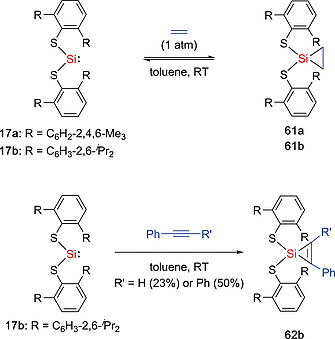
Equilibrium reactions with ethylene and activation of alkynes by acyclic silylenes **17a–b**.

Silylenes are well known to undergo cycloaddition reactions with unsaturated C–C bonds.^13^, ^43^ Similarly, silylenes **21**, **37a**, and **40** reacted with ethylene under mild conditions (1 atm, r.t.) to form the corresponding cycloaddition products **56**, **59**, and **60** (Scheme 16).^33^, ^40^, ^41^ In the case of silylene **15**, the reaction with ethylene at ambient temperatures gave the silirane product Si{CH_2_–CH_2_}{NDipp(SiMe_3_)}{Si(SiMe_3_)_3_} (**57**) in high yields. Furthermore, when compound **57** was heated to 60 °C under an ethylene atmosphere, an exceptional insertion of ethylene into Si–Si bond occurred to yield the modified silirane Si{CH_2_–CH_2_}{NDipp(SiMe_3_)}{CH_2_–CH_2_–Si(SiMe_3_)_3_} (**58**).^58^ A NMR experiment with deuterated ethylene indicated that the reaction proceeds via migratory insertion of the coordinated ethylene into the Si–Si bond, followed by the formation of the silirane with a C_2_D_4_ molecule. The groups of Power and Tuononen found that silylenes **17a** and **17b** also react with ethylene or alkynes to afford the [1+2] cycloaddition products **61a**, **61b**, and **62b** (Scheme 17).^59^ Interestingly, the ethylene addition products **61a** and **61b** were found to undergo reversible reactions with ethylene under ambient conditions. Notably, while many main‐group compounds can react with ethylene under mild conditions, the reversible reaction, which is a key step in catalytic cycles, remains rare.^60^ Products **61a** and **61b** were characterized using NMR spectroscopy (**61a** and **61b**) and X‐ray crystallographic analysis (**61b**). Van't Hoff analysis of the association of ethylene with **17b**, as determined by variable‐temperature ^1^H NMR spectroscopy, revealed a small value of Gibbs free energy (Δ*G*_assn_ = –24.9 kJ/mol at 300 K), which is comparably more favorable compared with that for the reaction of the phosphine supported Si(II) complex reported by the groups of Kato and Baceiredo (–3.0 kJ/mol).[60b] Similarly, the rare reversibility between Si(II) and Si(IV) compounds was found in silylenes **37a** and** 37b** which undergo an intermolecular insertion reaction into the C=C bond of the aromatic ligand framework to give silepins **38a** and** 38b**. The equilibrium between **37a** and **38a** was revealed by experimental and computational studies.

### 4.5 Activation of P_4_

White phosphorus (P_4_), which is easily obtained by the reduction of phosphate rock, is widely used as a starting material to synthesize organophosphorus compounds. In industrial processes, phosphorus chloride (PCl_n_) and phosphoryl chloride (POCl_3_) are precursors to organo‐phosphorus products are prepared by the chlorination or oxychlorination of white phosphorus.^61^ The eco‐friendly and atom efficient method of direct conversion of white phosphorus to phosphine containing products has also been considered.^62^ Currently, it was demonstrated that direct catalytic transformation of P_4_ into organo‐phosphorus compounds using a transition metal complex is possible.^63^ However, the stoichiometric and catalytic reaction of white phosphorus under mild conditions is challenging for both transition metal and main group compounds.

**Scheme 18:**
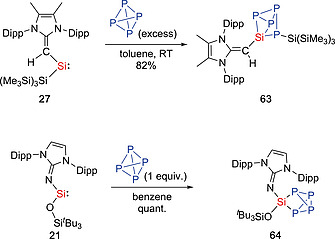
Activation of P_4_ by acyclic silylenes.

While several cyclic silylenes can react with P_4_, in most cases, the oxidative addition of a single P–P bond at the silicon center is observed,^64^ and the controlled reaction of P_4_ by main‐group compounds remains scarce.[61a], 65 The treatment of the vinyl(silyl)silylene **27** with P_4_ resulted in the formation of (^Me^IPrCH)Si(P_4_){Si(SiMe_3_)_3_} (**63**) (Scheme 18).^35^ It is plausible that compound **63** was formed by the oxidative addition of a P–P bond of P_4_ to silylene **27** with subsequent 1,2‐silyl migration. In this reaction, the cleavage of two P–P bonds of P_4_ and the regioselective formation of four new Si–P bonds were observed. In addition, imino(siloxy)silylene **21** was found to occur via the oxidative addition of 1 equivalent of P_4_ to give (IPrN)(^*t*^Bu_3_SiO)Si(P_4_) (**64**), which is different from that observed for the vinyl(silyl)silylene **27**.^49^

## 5. Summary and Outlook

Since the discovery of Jutzi's silicocene and Denk's NHSi, various cyclic silylenes and Lewis base stabilized silylenes have been reported. However, isolable two‐coordinate acyclic silylenes had been considered to be transient and non‐isolable compounds for a long time. The recent synthesis of stable acyclic silylenes has unlocked a new avenue in silicon chemistry, enabling metallomimetic behavior of this Earth‐abundant element. In this minireview, we mainly focused on the two‐coordinate acyclic silylene chemistry containing synthesis, properties, and application for small molecule activation. Acyclic silylenes bearing wide E–Si–E' angles and small HOMO–LUMO gaps are of extremely high reactivity which lead to the activation of important small molecules such as H_2_, NH_3_, ethylene, and CO_2_. Interestingly, while bis(silyl)silylene **40** exhibits a relatively large HOMO–LUMO gap (4.18 eV), **40** was found to occur the activation of H_2_. The singlet–triplet gap of **40** is comparatively small (10.5 kJ/mol). In addition, it was revealed that bis(arylthiolato)silylenes **17a** and **17b** show equilibrium reactions with ethylene at room temperature which is a key step in catalytic processes and is rare for silicon due to the unfavorable reduction of Si(IV) to Si(II).

These results for acyclic silylenes indicate the potential of main group compounds for future applications in the realms of catalytic and materials science. This study is inspiring for the molecular design of new silicon compounds which enable small molecule activation and the unprecedented cleavage of N–N bond in dinitrogen, N_2_. Acyclic silylenes are of extremely high reactivity due to the high‐energy lone‐pair on silicon (HOMO) and a low‐lying vacant *p*‐orbital (LUMO) which may interact with an empty π* orbital and an *n*‐orbital (a lone pair) on N_2_ leading to a weakening of the N–N bond. Furthermore, the hydrogenation and transfer hydrogenation of unsaturated molecules such as alkenes and alkynes mediated by acyclic silylenes is expected to be feasible. These studies imply that the steric and electronic tuning of the substituents enable the control of hydrogenation reactions. The accessibility of both Si(II) and Si(IV) oxidation states may lead to the utilization of silicon compounds in catalysts.
